# Meta-analysis to evaluate the comparative effectiveness of enzalutamide and abiraterone acetate for first-line treatment of metastatic castration-resistant prostate cancer in real-world settings

**DOI:** 10.3389/fonc.2025.1491314

**Published:** 2025-02-10

**Authors:** Armen Aprikian, Amit Bahl, Aurelius Omlin, Giulia Baciarello, Abhiroop Chakravarty, Prashanth Kondaparthi, Georgia Gourgioti, Thomas McLean, Alexis Serikoff, Andrew Chilelli

**Affiliations:** ^1^ Department of Oncology, McGill University, Montreal, QC, Canada; ^2^ Bristol Haematology and Oncology Centre, University Hospitals Bristol NHS Trust, Bristol, United Kingdom; ^3^ Onkozentrum Zurich, University of Zurich and Tumorzentrum Hirslanden Zurich, Zurich, Switzerland; ^4^ Azienda Ospedaliera San Camillo-Forlanini, Rome, Italy; ^5^ Parexel International, Hyderabad, India; ^6^ Astellas Pharma Europe, Addlestone, United Kingdom

**Keywords:** enzalutamide, abiraterone, prostatic neoplasms, meta-analysis, metastatic castration-resistant prostate cancer, overall survival, real-world evidence, real world

## Abstract

**Introduction:**

Androgen-receptor pathway inhibitors such as abiraterone and enzalutamide have demonstrated clinical benefit in patients with metastatic castration-resistant prostate cancer (mCRPC). The aim of this study was to conduct a meta-analysis of published real-world evidence studies comparing outcomes among patients treated with enzalutamide or abiraterone in the first-line setting.

**Methods:**

We conducted a systematic literature review to identify eligible studies. Evaluated outcomes were: overall survival (OS), progression-free survival, prostate-specific antigen (PSA) progression-free survival, PSA response, all-grade adverse events, grade ≥3 adverse events, treatment discontinuation, and dose reduction. Each outcome’s suitability for meta-analysis was evaluated by assessing whether there were sufficient data to make comparisons between studies, consistency between outcome definitions, and whether the studies adjusted for baseline patient characteristics. Outcomes deemed suitable for meta-analysis were analyzed using fixed-effect and random-effect models to obtain pooled-effect sizes. Sensitivity analyses were conducted to evaluate the robustness of conclusions.

**Results:**

Of 1849 records reviewed, 30 were eligible for inclusion. Most outcomes were deemed unsuitable for meta-analysis due to a lack of adjustment for baseline characteristics, issues with inconsistent outcome definitions, and the small number of studies reporting each outcome. The only outcome deemed suitable for meta-analysis was OS. A total of 17 studies reported hazard ratios (HRs) for OS, 11 of which were adjusted for baseline characteristics. Among the studies reporting adjusted HRs, the pooled-effect estimate favored enzalutamide over abiraterone (reference group) in the fixed-effect model (HR: 0.90 [95% CI: 0.87–0.93]) and the random-effect model (HR: 0.90 [95% CI: 0.86–0.94]). These results were consistent across all sensitivity analyses.

**Discussion:**

Across all analyses, enzalutamide demonstrated a statistically significant improvement in OS compared with abiraterone. These findings highlight the value of real-world evidence studies to demonstrate the potential of different therapies under real-world conditions and over long periods of time.

## 1 Introduction

Prostate cancer is the second-most commonly diagnosed cancer worldwide, and the fifth-highest cause of cancer-related deaths among men (1). Although prostate cancer is commonly managed using androgen-deprivation therapy (ADT), many patients experience disease progression resulting in prostate cancer that no longer fully responds to treatments that lower testosterone. Prostate cancer that progresses despite achieving castrate levels of testosterone is known as castration-resistant prostate cancer (CRPC), and may include metastases (metastatic CRPC [mCRPC]). Despite its reduced response to ADT, CRPC is still dependent on the androgen-receptor signaling pathway, which led to the development of androgen-receptor pathway inhibitors (ARPIs) for the treatment of CRPC (2). Results from several randomized controlled trials (RCTs) demonstrate that, compared with placebo, ARPIs can improve overall survival (OS), progression-free survival (PFS), and time to prostate-specific antigen (PSA) progression in patients with mCRPC (3–6). These benefits have been observed among patients with mCRPC who have previously been treated with docetaxel, in addition to those who are chemotherapy-naïve (3, 5–7). As a result of these positive outcomes, abiraterone and enzalutamide were approved for use in mCRPC (8, 9). However, as these treatments have primarily been evaluated during late-stage disease, there is a need for further evidence regarding the effectiveness and safety of these treatments.

Although RCTs are considered the gold standard for establishing the comparative efficacy of treatments, real-world evidence (RWE) studies may provide valuable information about treatment safety and effectiveness under real-world practice conditions (10, 11). These real-world analyses build on the findings of clinical trials and can help to elucidate treatment responses across the varied patient populations seen in real-world clinical practice. Moreover, RWE studies can enable head-to-head comparisons between treatments, something that is often unfeasible in the context of an RCT (12).

Over the past decade, multiple RWE studies have been conducted to compare the safety and effectiveness of abiraterone and enzalutamide across outcomes and geographies. While some RWE studies have favored enzalutamide in the first-line setting (13–16), others have favored abiraterone (17). Unfortunately, previous efforts to synthesize these findings via meta-analysis have been limited by insufficient data, heterogeneous patient populations, and inconsistent outcome definitions (18–20).

To bridge this evidence gap, the objective of the present study was to conduct a meta-analysis of published RWE studies that directly compared the effectiveness and safety of enzalutamide versus abiraterone for the first-line treatment of mCRPC. To address the issues with heterogeneity associated with previous meta-analyses, this analysis was limited to patients being treated with enzalutamide or abiraterone in the first-line mCRPC setting only and includes the most recent data available.

## 2 Methods

### 2.1 Systematic literature review

To identify the studies that would be eligible for meta-analysis, a systematic literature review of RWE studies comparing enzalutamide with abiraterone in the first-line treatment of mCRPC was conducted in line with the Preferred Reporting Items for Systematic Reviews and Meta-Analyses (PRISMA) methodology. Relevant articles were retrieved on May 22, 2023 from MEDLINE, Embase, and the Cochrane Database of Systematic Reviews. All publications available before May 22, 2023 were eligible for inclusion. Conference abstracts submitted to conferences held by the American Society of Clinical Oncology (ASCO), ASCO Genitourinary Cancers (ASCO GU), the American Urological Association (AUA), European Society for Medical Oncology (ESMO), European Association of Urology (EAU), and the International Society for Pharmacoeconomics and Outcomes Research (ISPOR) between 2019 and 2022 were eligible for inclusion. Full inclusion/exclusion criteria are presented in **Table 1** and the search strategy is presented in **Supplementary Table S1**.

**Table 1:** Study eligibility criteria for systematic literature review.

Elements	Inclusion criteria	Exclusion criteria
Population	• Age: adults (aged ≥18 years) • Disease: mCRPC	• Subpopulations with genetic mutations (e.g., homologous recombination repair)
Interventions and comparators	• Abiraterone • Enzalutamide • Administered in first-line setting (defined as the first treatment received for mCRPC)^a^	• Studies where the precise line of therapy cannot be determined
Outcomes^b^	• Effectiveness outcomes (e.g., OS, PFS, PSA-PFS, PSA response rate) • Safety outcomes • QoL outcomes	• Details in **Supplementary Material**
Study designs	• Cohort studies (retrospective/prospective) • Case-control studies	• Single-arm studies • Cross-sectional studies • Case studies/reports/series • RCTs
Language	• English language only	All other languages
Publication time frame	• No restriction	Not applicable

a A threshold of 90% was used to designate a study as first-line, which means if at least 90% of the study population was being treated first-line, the study was categorized as first-line.

b Outcomes were included in the literature review to evaluate their suitability for meta-analysis. Ultimately, only OS was deemed suitable for meta-analysis.

mCRPC, metastatic castration-resistant prostate cancer; OS, overall survival; PFS, progression-free survival; PSA, prostate-specific antigen; QoL, quality of life; RCT, randomized controlled trial.

Two independent reviewers conducted title and abstract screening, followed by full-text review. Disagreements were resolved by consensus. Data were extracted into an extraction grid by two independent reviewers. Risk of bias was assessed using the Newcastle–Ottawa Scale for assessing the quality of observational studies.

### 2.2 Meta-analysis

Effectiveness and safety outcomes considered for the meta-analysis were OS, PFS, PSA-PFS, PSA response, all-grade adverse events (AEs), grade ≥3 AEs, treatment discontinuation, and dose reduction. Each endpoint’s suitability for meta-analysis was evaluated by determining whether there were sufficient data to make direct comparisons between studies for each endpoint. Suitability for meta-analysis was also assessed by evaluating clinical heterogeneity between studies in terms of study and patient characteristics, study inclusion criteria, study design, and study outcome definitions. To ensure robust comparisons could be made between studies, an additional core consideration was whether data on each outcome were available for studies that adjusted for patient baseline characteristics. If outcome data from studies that adjusted for baseline characteristics were insufficient and/or substantial heterogeneity existed between studies, the outcome was deemed not suitable for meta-analysis. Fixed-effect and random-effect models were used to obtain pooled-effect sizes across studies. Full details of the fixed- and random-effect models are presented in the **Supplementary Methods**.

Heterogeneity between studies was assessed using the *I* ^2^ and the *τ* ^2^ statistics. Heterogeneity of *I* ^2^ ≤ 25% was considered low, *I* ^2^ > 25% and < 75% was considered moderate, and *I* ^2^ ≥ 75% was considered high (21). Publication bias was evaluated using Egger’s regression test.

Multiple sensitivity analyses were conducted to evaluate the robustness of the conclusions drawn from the meta-analysis. Sensitivity analyses were selected based on clinical expertise within the study team, who provided feedback on differences in baseline characteristics and advised on factors that may impact study results. Studies with notable differences in key patient characteristics were excluded in the sensitivity analyses, to assess any impact on the results. Sensitivity analyses were conducted using all eligible studies to ensure sufficient sample size for analysis. All analyses were conducted in R (version 4.3.1).

## 3 Results

### 3.1 Results of systematic literature review

**Figure 1:**
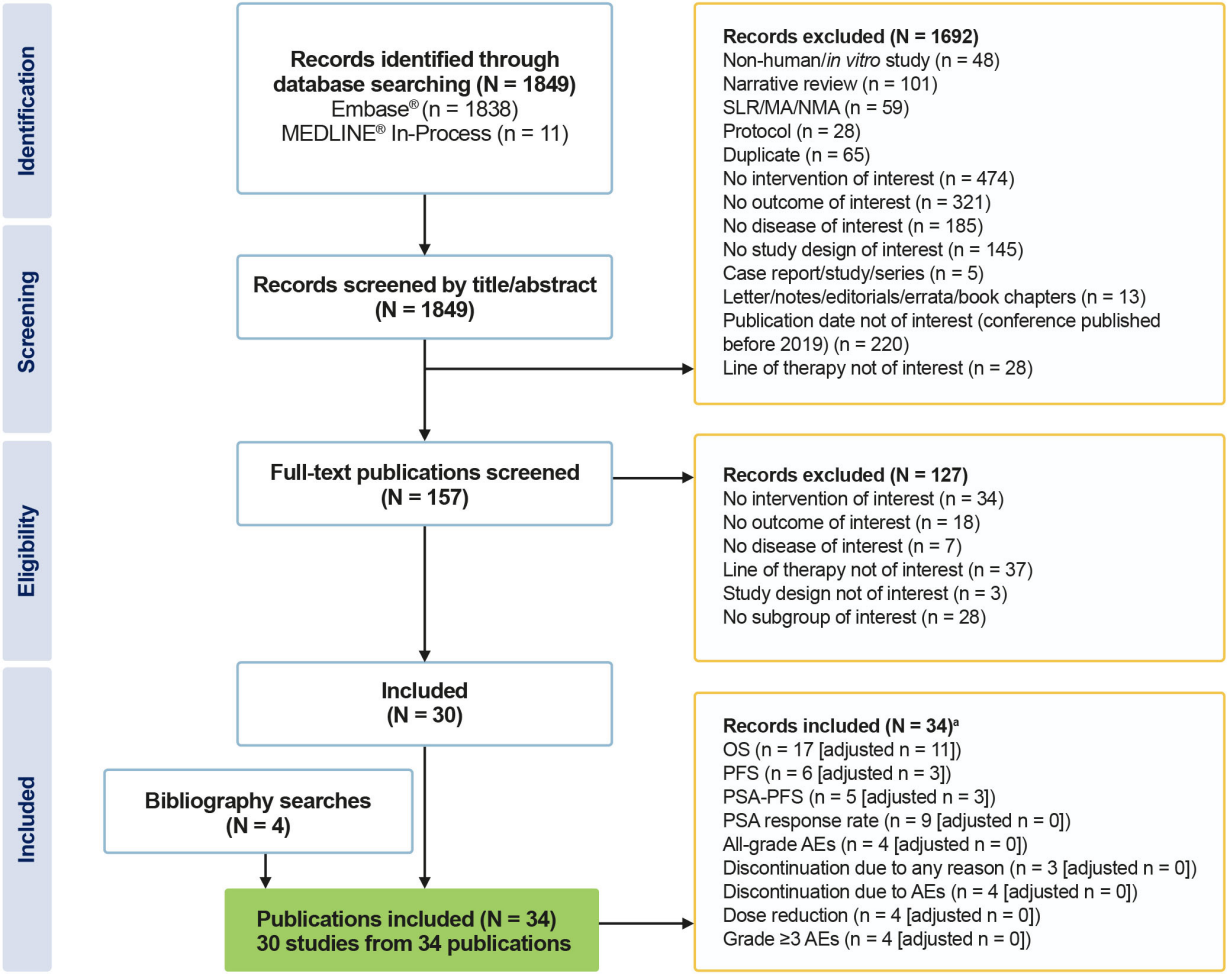
PRISMA flow diagram of included studies. ^a^Individual studies can report more than one outcome. MA, meta-analysis; NMA, network meta-analysis; PRISMA, Preferred Reporting Items for Systematic Reviews and Meta-Analyses; SLR, systematic literature review.

Of the 1849 records reviewed, 30 fulfilled the eligibility criteria for inclusion in the systematic review (**Figure 1**). All studies included patients with a confirmed diagnosis of mCRPC and no prior history of chemotherapy. Sample sizes ranged from 33 subjects to 10,308 subjects.

### 3.2 Assessment of outcome suitability for meta-analysis

Most outcomes were deemed unsuitable for meta-analysis due to a lack of adjustment for baseline characteristics, issues of inconsistent outcome definitions and assessment timings, and the small numbers of studies reporting each outcome. Studies that reported on each outcome that was deemed unsuitable for meta-analysis are presented in **Supplementary Table S2**. For the PFS outcome, there was a lack of consistency in how PFS was defined across studies, and only three of six studies adjusted for baseline characteristics. For PSA-PFS, only five studies reported hazard ratios (HRs), and only three of these adjusted for baseline characteristics. For PSA response rate, definitions were generally consistent, but only three of nine eligible studies included details on timing of assessment, and no studies adjusted for baseline characteristics. Few studies reported the treatment discontinuation or dose-reduction outcomes (n = 3 and n = 4, respectively), and none adjusted for baseline characteristics or provided definitions or details on timing of assessment. Finally, for AEs, there was a limited number of studies (n = 4 for all grades and n = 4 for grade ≥3), no studies adjusted for baseline patient characteristics between cohorts, and only two studies provided outcome definitions, of which only one provided details on the timing of assessment.

#### 3.2.1 Suitability of OS for meta-analysis

Overall survival was deemed suitable for meta-analysis due to the high number of studies reporting this outcome, the large number of studies that adjusted for baseline patient characteristics, and consistency in the endpoint definition. A total of 17 studies reported HRs for OS, 11 of which were adjusted for baseline characteristics. Nine studies adjusted for baseline patient characteristics using propensity score matching and inverse probability of treatment weighting, while two studies used multivariate Cox proportional hazard models. The relatively large number of eligible studies meant that primary, secondary, and several sensitivity analyses could be conducted to evaluate the robustness of the conclusions of the meta-analysis. The 17 eligible studies are presented in **Table 2**, and the detailed characteristics of those studies are presented in **Supplementary Table S3**.

**Table 2:** Studies reporting HRs for OS.

No.	Study name	Sample size	Adjusted^a^ (Y/N)	HR (95% CI)	Comparison
1	Marar et al., 2022^b^	3808	Y	1.21 (1.06–1.38)	ABI vs ENZA
2	Tagawa et al., 2021	3174	Y	0.84 (0.76–0.94)	ENZA vs ABI
3	Soleimani et al., 2021	270	Y	0.91 (0.70–1.19)	ABI vs ENZA
4	Scailteux et al., 2021	10,308	Y	0.90 (0.85–0.96)	ENZA vs ABI
5	Lopez-Campos et al., 2021	511	N	1.4 (1.04–1.89)	ABI vs ENZA
6	Carvajal et al., 2021^c^	100	N	1.27 (0.64–2.51)	ENZA vs ABI
7	Chowdhury et al., 2020	3003	Y	1.00 (0.79–1.27)	ABI vs ENZA
8	Komura et al., 2019	184	Y	0.86 (0.50–1.48)	ABI vs ENZA
9	Cesca et al., 2019	120	Y	0.66 (0.27–1.63)	ENZA vs ABI
10	Schoen et al., 2022	5822	Y	0.89 (0.84–0.95)	ENZA vs ABI
11	Chen et al., 2023	363	Y	0.68 (0.41–1.14)	ENZA vs ABI
12	An et al., 2023	3808	Y	0.96 (0.9–1.06)	ENZA vs ABI
13	Li et al., 2022	324	N	0.93 (0.54–1.62)	ENZA vs ABI
14	Alkan et al., 2021	134	N	0.87 (0.48–1.56)	ENZA vs ABI
15	Uchimoto et al., 2021	254	Y	1.55 (0.88–2.77)	ABI vs ENZA
16	Baillie et al., 2021	271	N	1.14 (0.68–1.91)	ENZA vs ABI
17	Oruc et al., 2021	191	N	0.77 (0.32–1.88)	ENZA vs ABI

a Studies considered “adjusted” were those that reported adjusting for baseline patient characteristics.

b HR for ABI vs ENZA for non-Hispanic White men.

c Carvajal et al., 2021 presented a Kaplan–Meier curve that was digitized to obtain pseudo-individual patient data followed by HR based on the Cox proportional hazards model.

ABI, abiraterone; CI, confidence interval; ENZA, enzalutamide; HR, hazard ratio; N, No; OS, overall survival; Y, Yes.

Of the studies that provided a definition for OS (n = 9), all but one study used the same definition (i.e., time from initiation of first-line treatment until death). Although eight studies did not report a definition, OS is a hard endpoint that is easily understood and measured and is likely to be the same or similar even in studies that did not report a definition.

### 3.3 Meta-analysis results for OS

**Figure 2:**
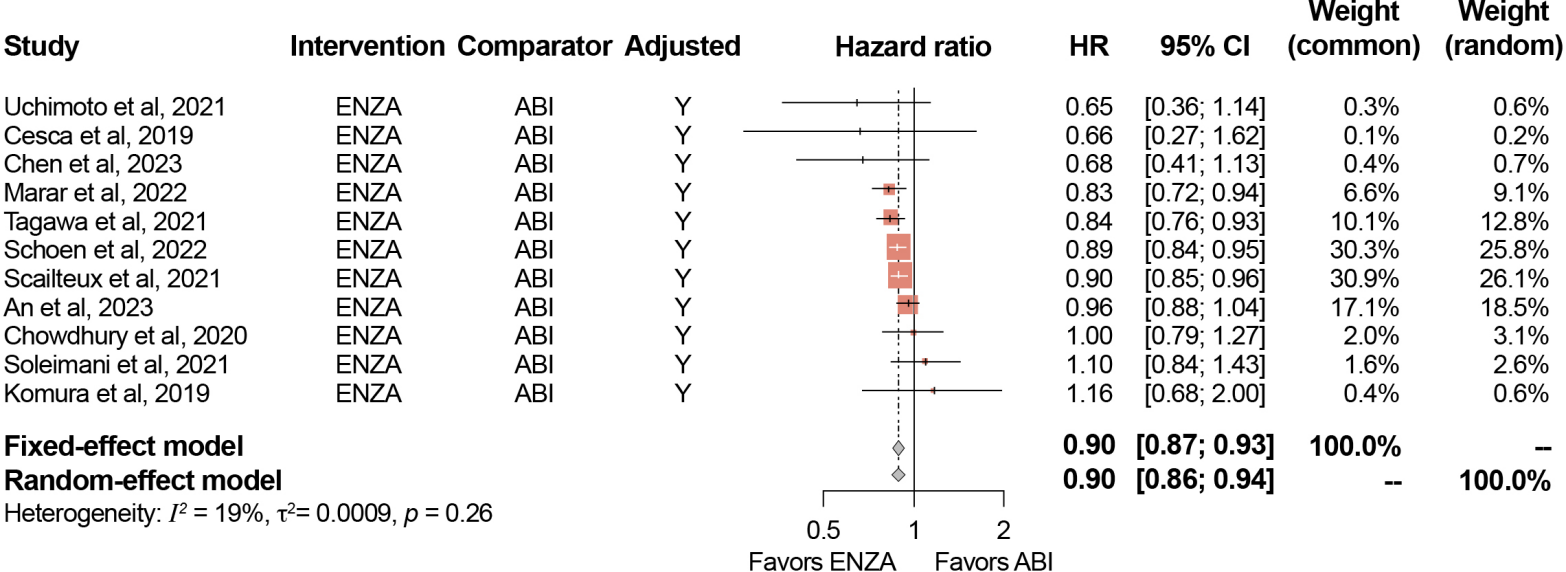
Meta-analysis results for OS (adjusted HRs). ABI, abiraterone; CI, confidence interval; ENZA, enzalutamide; HR, hazard ratio; Y, Yes. Minor differences between observed 95% CIs reported in study publications and estimated 95% CIs in meta-analysis results for certain studies are due to rounding in the reverse computation of upper and lower 95% CIs from standard error.

The primary analysis included the 11 studies reporting adjusted HRs for OS (i.e., those that adjusted for baseline patient characteristics), while the full 17 eligible studies were evaluated in a secondary analysis. Among the studies that reported adjusted HRs, the pooled-effect estimate favored enzalutamide over abiraterone (reference group) in both the fixed-effect model (0.90; 95% [confidence interval (CI)]: 0.87–0.93) and random-effect model (0.90; 95% CI: 0.86–0.94) (**Figure 2**), which were both statistically significant. The secondary analysis resulted in a similar statistically significant pooled effect (fixed-effect model: 0.90; 95% CI: 0.87–0.93; random-effect model: 0.90; 95% CI: 0.87–0.93) (**Table 3**).

**Table 3:** Results of primary, secondary, and sensitivity analyses for OS.

Scenario		Random-effect model ENZA vs ABI (Ref)	Fixed-effect model ENZA vs ABI (Ref)
Primary analysis: Studies that adjusted for baseline characteristics (n = 11)		0.90 (0.86–0.94)	0.90 (0.87–0.93)
Secondary analysis: Include studies that did not adjust for baseline characteristics (n = 17)		0.90 (0.87–0.93)	0.90 (0.87–0.93)
Sensitivity analyses (based on n = 17 studies)			
1	Exclude studies with a high percentage of subjects with ECOG PS ≥2 (Alkan et al., 2021 and Oruc et al., 2021 excluded)	0.90 (0.86–0.94)	0.90 (0.87–0.93)
2	Exclude studies with notable differences between ENZA and ABI in terms of ECOG PS (Baillie et al., 2021 and Miyake et al., 2017 excluded)	0.90 (0.86–0.93)	0.90 (0.87–0.93)
3	Exclude studies with geriatric population (mean age >80 years) (Soleimani et al., 2021 excluded)	0.89 (0.87–0.93)	0.89 (0.87–0.93)
4	Notable difference in diabetes prevalence between ENZA and ABI arms (Carvajal et al., 2021 excluded)	0.90 (0.86–0.93)	0.90 (0.87–0.93)
5	Exclude studies with differences in percentage or type of concomitant medication between ENZA and ABI arms (Shore et al., 2019 and Chen et al., 2023 excluded)	0.90 (0.87–0.93)	0.90 (0.87–0.93)
6	Exclude studies with notable differences between ENZA and ABI arms in terms of liver disease and in lymph node metastases (Chen et al., 2023 excluded)	0.90 (0.87–0.93)	0.90 (0.87–0.93)
7	Exclude studies with notable differences in baseline PSA value (Chowdhury et al., 2020, Tagawa et al., 2021, Alkan et al., 2021, Komura et al., 2019, and Carvajal et al., 2021 excluded)	0.90 (0.86–0.94)	0.90 (0.87–0.93)
8	Exclude all studies with notable differences in baseline characteristics (combining scenarios 1–7)	0.90 (0.86–0.94)	0.90 (0.87–0.93)
9	Exclude studies with sample size <500	0.89 (0.85–0.93)	0.89 (0.86–0.93)
10	Exclude studies with prior docetaxel usage (Schoen et al., 2022 excluded)	0.90 (0.86–0.94)	0.90 (0.87–0.94)
11	Exclude studies with low Newcastle–Ottawa score, indicating poor methodology (Shore et al., 2019 excluded)	0.90 (0.87–0.93)	0.90 (0.87–0.93)
12	Include unadjusted HR from studies reporting both adjusted and unadjusted HR^a^	0.89 (0.86–0.93)	0.89 (0.86–0.93)

a “Adjusted HR” refers to HRs that were adjusted for baseline patient characteristics; “unadjusted HR” refers to HRs that were not adjusted for baseline patient characteristics.

ABI, abiraterone; CI, confidence interval; ECOG PS, Eastern Cooperative Oncology Group Performance Status; ENZA, enzalutamide; HR, hazard ratio; OS, overall survival; PSA, prostate-specific antigen; Ref, reference.

The results of the sensitivity analyses conducted are presented in **Table 3**. Across all 12 sensitivity analyses, pooled estimates in the fixed-effect and random-effect models ranged from 0.89–0.90 and were all statistically significant. Detailed results of the sensitivity analyses are presented in the **Supplementary Material**.

#### 3.3.1 Heterogeneity assessment

For studies only reporting adjusted HRs for OS, *I* ^2^ was 19% (95% CI: 0–59.2%) and τ was 0.03 (95% CI: 0–0.18; p=0.26). For the analysis that included both adjusted and unadjusted HRs, *I* ^2^ was 4% (95% CI: 0–52.9%) and τ was 0.01 (95% CI: 0–0.14 p=0.41), suggesting low heterogeneity. These results suggest that although the directionality of the heterogeneity was similar, the data in the studies reporting adjusted results were slightly more heterogeneous than the data in the studies reporting unadjusted results.

#### 3.3.2 Publication bias

**Figure 3:**
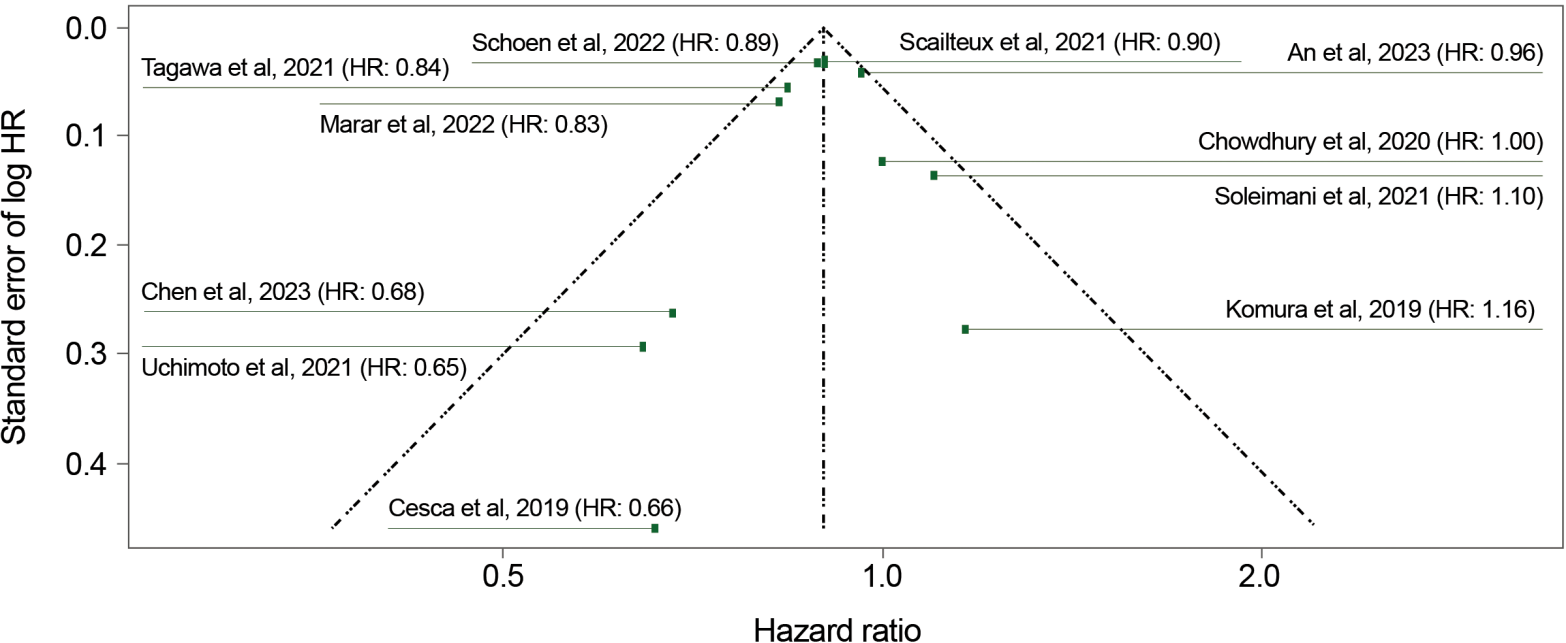
Funnel plot for overall survival (adjusted HRs). HR, hazard ratio. Linear regression test of funnel plot asymmetry: Test result: τ = −0.31, df = 9, p = 0.76. Sample estimates: bias = −0.18; se.bias = 0.56; intercept = −0.10; se.intercept = 0.03.

Publication bias was close to zero for both the analysis of adjusted HRs (−0.18; standard error [SE], 0.56; p=0.76) (**Figure 3**) and for the analysis of both adjusted and unadjusted HRs (−0.05; SE, 0.36; p=0.87). These findings indicate an absence of funnel plot asymmetry and publication bias.

## 4 Discussion

In this meta-analysis of RWE, enzalutamide showed a statistically significant OS benefit when compared with abiraterone among patients with mCRPC in the first-line setting. The pooled-effect size and statistical significance were consistent across fixed-effect and random-effect models in the primary analysis, the secondary analysis, and across 12 different sensitivity analyses. This robustness strengthens our confidence in these conclusions. Moreover, a study by George and colleagues, published subsequent to this meta-analysis, found an identical magnitude of effect, lending further credibility to our findings (22).

Our assessments of other effectiveness and safety endpoints in real-world settings suggested that meta-analysis was not feasible for outcomes other than OS due to the frequent lack of adjustment for baseline patient characteristics, the small number of studies reporting each outcome, different endpoint definitions used, and the different methods and timing of assessment. As differences in baseline characteristics may impact outcomes (e.g., comorbid conditions) (23), future real-world analyses should ensure methods of adjustment are applied when making comparative assessments. In terms of outcome definitions, we identified substantial differences in how endpoints were defined and measured across all outcomes except OS. These findings are aligned with a 2022 scoping review by Shah et al., which noted that efforts to conduct meta-analyses are made more challenging due to the wide range of outcome definitions and contexts (19). Similarly, Hettle et al. (2023) noted substantial heterogeneity in how PFS was defined across RWE studies in mCRPC (24). These findings support the need for consistency in outcome definitions, particularly when there can be multiple ways of interpreting an outcome or when different clinical thresholds and timings can be used to evaluate an outcome. Guidelines such as those developed by the Prostate Cancer Clinical Trials Working Group 3 provide recommendations for how endpoints should be defined in clinical trials (25), and similar guidelines for ensuring consistent outcome definitions in RWE studies would be beneficial for enabling analyses of outcomes other than OS in the future.

The treatment landscape for prostate cancer is rapidly evolving and new data are regularly emerging on the effectiveness of ARPIs. While ARPIs are recommended across the prostate cancer treatment spectrum, including for the clinical management of metastatic hormone-sensitive prostate cancer, mCRPC, and non-metastatic CRPC, evidence suggests that in real-world clinical practice, physicians most commonly continue to prescribe abiraterone and enzalutamide in mCRPC (26), and many patients do not receive intensified treatment, despite clinical guidelines recommending this approach (27, 28). These challenges illustrate the ongoing need for aggregated data with longer follow-up periods that permit sufficient time for longer-term outcomes to develop and be evaluated across treatment regimens. Moreover, our findings highlight the importance of standardized outcome definitions in RWE studies across disease states to enable meta-analyses that can inform treatment decisions in real-world settings, regardless of how the treatment landscape evolves.

### 4.1 Strengths and limitations

This study had several strengths. It is among the first to compare enzalutamide and abiraterone across multiple RWE studies, including those that adjusted for baseline patient characteristics and those that did not. In addition, this study benefits from its inclusion of data from multiple countries and datasets, including the most recent and largest RWE studies available. The inclusion of these data permitted the meta-analysis of OS, which was not previously possible. In addition, because a large number of studies were available for the analysis of OS, multiple sensitivity analyses could be conducted, lending credibility to our findings. Finally, the majority of studies reporting OS data adjusted for baseline patient characteristics, permitting more robust conclusions to be drawn from the head-to-head comparison of enzalutamide and abiraterone.

This study also has limitations. First, although our primary analysis focused on studies that adjusted for baseline patient characteristics, not all characteristics were measured, and therefore could not be controlled for in the analysis. We attempted to account for the possibility of residual confounding by conducting multiple sensitivity analyses which excluded studies that may have been impacted by differences in baseline characteristics. The results of all these sensitivity analyses were consistent with our primary results. Second, the studies included in our analyses focused only on OS relative to first-line treatment; they did not account for the potential role of subsequent-line therapies. Although this was a study of first-line therapies, subsequent-line therapies can confound OS results, and previous studies have recommended that these therapies be reported (29, 30), but these data were not available in our analysis. Third, although our evaluation of heterogeneity found that it was low for the outcome of OS, future studies should attempt to address the heterogeneity in evaluations of other effectiveness and safety outcomes. Fourth, many of the studies included in our analysis had relatively small sample sizes. We accounted for this by conducting a sensitivity analysis that excluded all studies with a sample size of fewer than 500 participants. The results of this sensitivity analysis were consistent with our primary analysis. Fifth, systematic literature reviews and meta-analyses are susceptible to publication bias. We sought to address this limitation using Egger’s regression test, which suggested a low risk of publication bias in our analysis. Sixth, a limitation of meta-analyses is the possibility of overlapping cohorts. In our analysis, this issue primarily affected two of the included studies (Tagawa et al. and Schoen et al.), which included partially overlapping patient cohorts. As the datasets, data maturity, and adjustment strategies in each study differed, we concluded that the benefit of keeping both studies in the primary analysis outweighed the potential biases introduced by the overlapping cohorts. Nonetheless, we also conducted sensitivity analyses that excluded these studies and found no difference in HR compared with our primary analysis. Lastly, our analysis relied on real-world data, which are susceptible to bias, heterogeneity, and measurement errors. Our use of sensitivity analyses sought to address this issue by excluding studies with notable differences in baseline characteristics. However, the substantial differences in outcome definitions between studies meant that we were unable to analyze most endpoints of interest.

## 5 Conclusion

This study combines over a decade of data from a range of jurisdictions, allowing us to compare the effectiveness of enzalutamide versus abiraterone on OS in real-world settings. Across studies and analyses, enzalutamide demonstrated a statistically significant improvement in OS compared with abiraterone for first-line treatment of mCRPC. These findings highlight the value of RWE studies to demonstrate the potential of different therapies under real-world conditions and over long periods of time, especially in situations where no RCTs comparing specific therapies are available.

## Data Availability

The original contributions presented in the study are included in the article/**Supplementary Material**. Further inquiries can be directed to the corresponding author.
