# Supplementary material for: Meta-analysis to evaluate the comparative effectiveness of enzalutamide and abiraterone acetate for first-line treatment of metastatic castration-resistant prostate cancer in real-world settings
Source: Front Oncol. 2025 Feb 10;15:1491314. doi: 10.3389/fonc.2025.1491314 (PMC11849621; doi:10.3389/fonc.2025.1491314)
Supplement: Supplementary file 1 [file DataSheet1.docx]

# Supplementary Material

## Supplementary Table 1. Embase and MEDLINE search strategy

| **#ID** | **Searches** | **Hits** |
| --- | --- | --- |
| 1 | 'metastatic castration resistant prostate cancer'/exp OR 'metastatic castration resistant prostate cancer' OR 'metastatic castration-resistant prostate cancer' OR 'metasta* crpc' OR mcrpc OR 'prostate cancer'/exp OR 'prostate cancer' OR 'prostate carcinoma'/exp OR 'prostate carcinoma' OR 'prostate tumor'/exp OR 'prostate tumor' | 319,850 |
| 2 | (metasta* NEAR/4 (prostate OR prostatic OR prostat*) NEAR/4 (cancer OR neoplas$ OR malignan* OR carcinoma OR tumor* OR tumour* OR cancer* OR tumor OR tumors OR tumour OR tumours)):ti,ab | 33,308 |
| 3 | 'castration resistant':ti,ab OR 'castration-resistant':ti,ab OR 'hormone-resistant':ti,ab | 18,910 |
| 4 | ((castrat* OR hormon*) NEAR/4 (refractory OR resistant)):ti,ab | 26,717 |
| 5 | #3 OR #4 | 26,717 |
| 6 | #2 AND #5 | 12,782 |
| 7 | #1 OR #6 | 319,853 |
| 8 | 'enzalutamide'/exp | 9549 |
| 9 | enzalutamide:ti,ab,kw | 6110 |
| 10 | xtandi*:ti,ab,kw OR enzalutamid*:ti,ab,kw OR 'mdv 3100':ti,ab,kw OR mdv3100:ti,ab,kw OR 'asp 9785':ti,ab,kw OR asp9785:ti,ab,kw | 6441 |
| 11 | #8 OR #9 OR #10 | 10,159 |
| 12 | 'abiraterone'/exp | 6568 |
| 13 | 'abiraterone acetate'/exp | 3616 |
| 14 | abiraterone:ti,ab,kw OR zytiga$:ti,ab,kw OR zaitiga:ti,ab,kw OR zaytiga:ti,ab,kw OR yonsa:ti,ab,kw OR (abirateron*AND acetat*):ti,ab,kw OR zytiga:ti,ab,kw OR abiratas:ti,ab,kw OR abretone:ti,ab,kw OR abirapro:ti,ab,kw OR (cb AND 7630) OR cb7630:ti,ab,kw OR 'cb 7630':ti,ab,kw OR abirateron*:ti,ab,kw | 6571 |
| 15 | #12 OR #13 OR #14 | 10,192 |
| 16 | #11 AND #15 | 6171 |
| 17 | #7 AND #16 | 5971 |
| 18 | 'observational study'/exp OR 'cohort analysis'/exp | 1,245,424 |
| 19 | 'observational':ti,ab,kw OR cohort*:ti,ab,kw OR 'longitudinal':ti,ab,kw OR 'retrospective':ti,ab,kw OR 'prospective':ti,ab,kw | 3,548,707 |
| 20 | #18 OR #19 | 3,752,751 |
| 21 | #17 AND #20 NOT [23-05-2023]/sd | 1838 |

## Supplementary Table 2. Studies reporting outcomes that were not suitable for meta-analysis

| **Studies reporting HRs for PFS** | | | | | | |
| --- | --- | --- | --- | --- | --- | --- |
| **No.** | **Study name** | | **Adjusted (Y/N)^a^** | **HR (95% CI)** | | **Comparison** |
| 1 | Cesca et al. 2019 | | Y | 0.36 (0.20–0.64) | | ENZA vs ABI |
| 2 | Soleimani et al. 2021 | | Y | 0.66 (0.50–0.88) | | ENZA vs ABI |
| 3 | Chowdhury et al. 2020 | | Y | 1.040 (0.851–1.271) | | ABI vs ENZA |
| 4 | Matsubara et al. 2018 | | N | 0.80 (0.54–1.21) | | ABI vs ENZA |
| 5 | Alkan et al. 2021^b^ | | N | 1.66 (0.92–3.02) | | ENZA vs ABI |
| 6 | Oruc et al. 2021 | | N | 0.94 (0.45–1.96) | | ABI vs ENZA |
| **Studies reporting HRs for PSA-PFS** | | | | | | |
| **No.** | **Study name** | | **Adjusted (Y/N)^a^** | **HR (95% CI)** | | **Comparison** |
| 1 | Terada et al. 2017 | | N | 0.88 (0.66–1.19) | | ABI vs ENZA |
| 2 | Soleimani et al. 2021 | | Y | 0.73 (0.53–1.01) | | ENZA vs ABI |
| 3 | Miyake et al. 2017 | | N | 2.51 (1.02–6.12)^c^ | | ABI vs ENZA |
| 4 | Komura et al. 2019 | | Y | 1.03 (0.67–1.57) | | ENZA vs ABI |
| 5 (1) | Chen et al. 2023 (adjusted) | | Y | 0.86 (0.63–1.17) | | ENZA vs ABI |
| 5 (2) | Chen et al. 2023 (crude) | | N | 0.89 (0.66–1.2) | | ENZA vs ABI |
| 5 (3) | Chen et al. 2023 (MACE) | | N | 0.20 (0.07–0.55) | | ENZA vs ABI |
| **Studies reporting PSA response rate data** | | | | | | |
| **No.** | **Study name** | **Adjusted (Y/N)^a^** | **ENZA rate (%)** | | **ABI rate (%)** | |
| 1 | Soleimani et al. 2021 | N | 80/125 (64) | | 69/153 (45) | |
| 2 | Ferriero et al. 2019 | N | 47/49 (96) | | 59/88 (67) | |
| 3 | Miyake et al. 2017 | N | 118/167 (71) | | 60/113 (53) | |
| 4 | Komura et al. 2019^d^ | N | 60/92 (65) | | 54/92 (59) | |
| 5 | Terada et al. 2017 | N | 44/80 (55) | | 53/110 (48) | |
| 6 | Matsubara et al. 2018 | N | 24/47 (51) | | 24/50 (48) | |
| 7 | Chen et al. 2023 | N | 119/157 (76) | | 131/206 (64) | |
| 8 | Del Rosario García et al. 2021^e^ | N | 20/25 (80) | | 13/17 (76) | |
| 9 | Alkan et al. 2021^d^ | N | 40/57 (70) | | 56/77 (73) | |
| **Studies reporting rate of adverse events** | | | | | | |
| **No.** | **Study name** | **Adjusted (Y/N)** | **ENZA rate (%)** | | **ABI rate (%)** | |
| 1 | Shore et al. 2019 | N | 26/50 (52) | | 18/50 (36) | |
| 2 | Thiery-Vuillemin et al. 2020 | N | 82/106 (77) | | 72/105 (69) | |
| 3 | Ferreiro et al. 2020 | N | 8/49 (16) | | 9/88 (10) | |
| 4 | Miyake et al. 2017 | N | 125/167 (75) | | 75/113 (66) | |
| **Studies reporting treatment discontinuation** | | | | | | |
| **No.** | **Study name** | **Adjusted (Y/N)** | **ENZA rate (%)** | | **ABI rate (%)** | |
| 1 | Thiery-Vuillemin et al. 2020 | N | 38/106 (36) | | 42/105 (40) | |
| 2 | Soleimani et al. 2021 | N | 51/125 (41) | | 44/153 (29) | |
| 3 | Chowdhury et al. 2020 | N | 171/227 (75) | | 607/754 (81) | |
| **Studies reporting dose reduction** | | | | | | |
| **No.** | **Study name** | **Adjusted (Y/N)** | **ENZA rate (%)** | | **ABI rate (%)** | |
| 1 | Shore et al. 2019 | N | 8/50 (16) | | 3/50 (6) | |
| 2 | Soleimani et al. 2021 | N | 56/125 (45) | | 35/153 (23) | |
| 3 | Alkan et al. 2021 | N | 0/57 (0) | | 2/77 (3) | |
| **Studies reporting adverse events grade ≥3** | | | | | | |
| **No.** | **Study name** | **Adjusted (Y/N)** | **ENZA rate (%)** | | **ABI rate (%)** | |
| 1 | Shore et al. 2019 | N | 2/50 (4) | | 3/50 (6) | |
| 2 | Miyake et al. 2017 | N | 19/167 (11) | | 5/113 (4) | |
| 3 | Ferreiro et al. 2020 | N | 1/49 (2) | | 0/88 (0) | |
| 4 | Alkan et al. 2021 | N | 1/57 (2) | | 7/77 (9) | |
| **Studies reporting adverse events leading to treatment discontinuation** | | | | | | |
| **No.** | **Study name** | **Adjusted (Y/N)** | **ENZA rate (%)** | | **ABI rate (%)** | |
| 1 | Shore et al. 2019 | N | 2/50 (4) | | 2/50 (4) | |
| 2 | Thiery-Vuillemin et al. 2020 | N | 12/106 (11) | | 5/105 (5) | |
| 3 | Alkan et al. 2021 | N | 3/57 (6) | | 6/77 (8) | |
| 4 | Chowdhury et al. 2020 | N | 23/227 (10) | | 43/754 (6) | |

^a^ Studies noted as “adjusted” are those that reported adjustment for baseline patient characteristics.

^b^ Alkan et al. 2021 presented a Kaplan–Meier curve that was digitized to obtain pseudo-individual patient data followed by HR based on the Cox proportional hazards model.

^c^ 95% CI is obtained using the p-value of 0.043 reported in the univariate analysis using treatment as covariate.

^d^ Raw patient numbers used to obtain rate estimates.

^e^ Minor differences in percentages presented in the table and those found in the original publication are due to rounding when back calculating event rates.

Abbreviations: ABI, abiraterone; CI, confidence interval; ENZA, enzalutamide; HR, hazard ratio; MACE, major adverse cardiovascular event; PFS, progression-free survival; PSA, prostate-specific antigen.

## Supplementary Table 3. Characteristics of studies that evaluated OS between treatment groups and reported HRs

| Study ID, country | Treatment group | Sample size | Median follow-up period (months) | Evaluated population | SD | OS median (months) | IQR (lower–upper) | 95% CI (lower–upper) | HR | 95% CI (lower–upper) | p-value |
| --- | --- | --- | --- | --- | --- | --- | --- | --- | --- | --- | --- |
| Briones Carvajal et al. 2021, Canada (1),^a^ | ABI | 100 | 13.7 | 50 | NR | 35.7 | 20.4–52.5 | NR | Ref | NR | NR |
|  | ENZA |  | 19.5 | 50 | NR | 34 | 25.7–38.0 | NR | 1.27 | 0.64–2.51 | NR |
| López-Campos et al. 2021 (2),^b^ | ABI | 511 | 30.2 | 391 | NR | 29 | NR | NR | 1.4 | 1.039–1.887^c^ | p = 0.027 |
|  | ENZA |  |  | 120 | NR | 38.1 | NR | NR | Ref | Ref | NR |
| Soleimani et al. 2021, Canada (3) | ABI | 270 | NR | 150 | NR | NR | NR | NR | Adjusted: 0.91 | Adjusted: 0.7–1.19 | p = 0.48 |
|  | ENZA |  | NR | 120 | NR | NR | NR | NR | Ref | Ref | Ref |
| Tagawa et al. 2021, USA (4)  (George et al. 2020, Ramaswamy et al. 2019) (5, 6) | ABI | 3174 | 19.07 | 1945 | NR | 25.87 | NR | NR | Ref | Ref | Ref |
|  | ENZA |  | 18.27 | 1229 | NR | 29.63 | NR | NR | Adjusted: 0.84 | Adjusted: 0.76–0.94 | p = 0.0012 |
| Scailteux et al. 2021, France (7) | ABI | 10,308 | 23.2 | 6585 | NR | 31.7 | NR | NR | Ref | Ref | NR |
|  | ENZA |  | 20.8 | 3723 | NR | 34.2 | NR | NR | Adjusted: 0.90 | Adjusted: 0.85–0.96 | NR |
| Chowdhury et al. 2020, multiple countries (8) | ABI | 3003 | 18.6 | 754 | NR | 27.1 | NR | 25.3–28.9 | Adjusted: 1.000  Unadjusted: 0.983 | Adjusted: 0.788–1.27  Unadjusted: 0.798–1.211 | Adjusted:  p = 0.986  Unadjusted: p = 0.8741 |
|  | ENZA |  |  | 227 | NR | 27.1 | NR | 21.8–32.7 | Ref | Ref | Ref |
| Cesca et al. 2019, Brazil (9) | ABI | 120 | 21.2 | 101 | NR | 36.1 | NR | NR | Ref | Ref | Ref |
|  | ENZA |  |  | 43 | NR | Not reached | NR | NR | Adjusted: 0.66  Unadjusted: 0.60 | Adjusted: 0.27–1.63  Unadjusted: 0.27–1.36 | Adjusted:  p = 0.37  Unadjusted: p = 0.22 |
| Komura et al. 2019, Japan and USA (10) | ABI | 184 | NR | 92 | NR | 42 | NR | NR | Adjusted: 0.86 | Adjusted: 0.5–1.48 | p = 0.58 |
|  | ENZA |  | NR | 92 | NR | 38 | NR | NR | Ref | Ref | Ref |
| Schoen et al. 2023, USA (11) | ABI | 5822 | 23.2 | 3318 | NR | 22.1 | NR | NR | Ref | Ref | Ref |
|  | ENZA |  |  | 2504 | NR | 24.2 | NR | NR | Adjusted: 0.89 | Adjusted: 0.84–0.95 | p = 0.001 |
| Chen et al. 2023, Taiwan (12) | ABI | 363 | Mean: 17.33 | 206 | NR | NR | NR | NR | Ref | Ref | NR |
|  | ENZA |  | Mean: 20.75 | 157 | NR | NR | NR | NR | Adjusted: 0.68  Unadjusted: 0.68 | Adjusted: 0.41–1.14  Unadjusted: 0.42–1.11 | NR |
| An et al. 2023, USA (13) | ABI | 3808 | NR | 1457 | NR | NR | NR | NR | Ref | Ref | NR |
|  | ENZA |  |  | 1278 | NR | NR | NR | NR | Adjusted: 0.96  Unadjusted: 0.96 | Adjusted: 0.90–1.06  Unadjusted: 0.87–1.06 | NR |
| Li et al. 2022, Taiwan (14) | ABI | 324 | 28.91 | 81 | NR | NR | NR | NR | Ref | Ref | Ref |
|  | ENZA |  |  | 64 | NR | NR | NR | NR | Univariate analysis: 0.93 | 0.54–1.62 | p = 0.803 |
|  | ABI |  |  | 81 | NR | NR | NR | NR | Ref | Ref | Ref |
|  | ENZA |  |  | 64 | NR | NR | NR | NR | Multivariate analysis: 1.39 | 0.44–4.34 | p = 0.572 |
| Alkan et al. 2021, Turkey (15) | ABI | 134 | NR | 77 | NR | 18 | NR | 15.2–20.7 | Ref | Ref | Ref |
|  | ENZA |  |  | 57 | NR | 20 | NR | 4.4–35.5 | 0.87 | 0.48–1.56 | p = 0.65 |
| Marar et al. 2022, USA (16) | ABI | 3808 | 13 | 1202 (non-Hispanic White) | NR | 17 | NR | IQR: 9–32 | Adjusted: 1.21 | Adjusted: 1.06–1.38 | NR |
|  | ENZA |  |  |  | NR | 20 | NR | IQR: 10–36 | Ref | Ref | NR |
|  | ABI |  |  | 170 (African American men) | NR | 24 | NR | IQR: 11–37 | Adjusted: 1.05 | Adjusted: 0.74–1.5 | NR |
|  | ENZA |  |  |  | NR | 24 | NR | IQR: 13–37 | Ref | Ref | NR |
| Uchimoto et al. 2021, Japan (17) | ABI | 254 | NR | 119 | NR | NR | NR | NR | Multivariate adjusted: 1.55 | Multivariate adjusted: 0.88–2.77 | p = 0.119 |
|  | ENZA |  |  | 135 | NR | 45 | NR | NR | Ref | Ref | Ref |
| Baillie et al. 2020, Scotland (18) | ABI | 271 | 16.3 | 63 | NR | 20.9 | NR | 14.9–29 | Ref | Ref | Ref |
|  | ENZA |  | 20.7 | 42 | NR | 16 | NR | 9.8–NR | Univariate unadjusted analysis: 1.14 | Univariate unadjusted analysis: 0.68– 1.91 | p = 0.63 |
| Oruç et al. 2021, Turkey (19) | ABI | 191 | 11.3 | 27 (visceral metastasis) | NR | 6 | NR | 1.8–10.1 | Ref | Ref | Ref |
|  | ENZA |  |  | 18 (visceral metastasis) | NR | 11 | NR | 0.9–23.1 | 0.77 | 0.32–1.88 | p = 0.58 |

“Adjusted” refers to HRs from cohorts that were adjusted for baseline patient characteristics.

^a^ Carvajal et al. presented a Kaplan–Meier curve in a poster that was digitized to obtain pseudo-individual patient data followed by HR based on the Cox proportional hazards model.

^b^ 95% CI is obtained using the p-value of 0.027 reported for the analysis using treatment as covariate.

Minor differences between observed 95% CIs reported in study publications (Table 2) and estimated 95% CIs in meta-analysis results (Figure 2) for certain studies are due to rounding in the reverse computation of upper and lower 95% CI from standard error.

^c^ Confidence interval calculated from p-value.

Abbreviations: ABI, abiraterone; CI, confidence interval; ENZA, enzalutamide; HR, hazard ratio; ID, identification; IQR, interquartile range; NR, not reported; OS, overall survival; Ref, reference; SD, standard deviation.

## Supplementary Methods

### Fixed-effect (FE) model

As explained earlier, an FE model assumes that the true effect size is not a variable across individual studies; instead, sampling error is, and thus a FE model is given by:

$$\hat{\theta}_{i}= \theta+\epsilon_{i}$$

where $\theta$ and $\epsilon_{i}$ are true effect size and sampling error for each study respectively.

To calculate pooled-effect size under the FE model, we obtained weighted average across all studies where the weights are defined as the inverse of the variance for each effect size across studies given by:

$w_{i}$ = $\frac{1}{s_{i}^{2}}$ ; $s_{i}^{2}$ is the square of standard error for each study

This also underlines the fact that studies with smaller standard error are allocated larger weights. This method is often listed as an inverse-variance meta-analysis. Estimate for $\hat{\theta}$ is given by:

$\hat{\theta}=\frac{\sum_{i=1}^{I} \hat{\theta}_{i}w_{i}}{\sum_{i=1}^{I} w_{i}}$; $\hat{\theta}^{'}s$ are the effect size for each study

For outcomes where we have binary-effect size data, such as responders vs non-responders, an alternative method called Mantel–Haenszel (20) is used to calculate the weighted average under the fixed-effect assumption.

### Random-effect (RE) model

With the RE model, we can account for the fact that the difference across individual studies should not be attributed only to sampling error but to some other source of variance as well. This burden of excess variance justifies that every individual study has some uniqueness and should not be considered a part of one single population. The RE model is given by:

$\hat{\theta}_{i}= \theta_{i}+\epsilon_{i}$*(1)*

$\theta_{i}= \mu+ \delta_{i}$ *(2)*

$\hat{\theta}_{i}= \mu+ \delta_{i}+\epsilon_{i}$ *(From 1 and 2)*

The RE model assumes that $\theta_{i}$ is true effect size per study, which is derived from a space of distribution of effect size with mean $\mu$ and error $\delta_{i}$.

To obtain pooled-effect size under the RE model, we apply an approach that is slightly different from the FE model. It is assumed in the RE model that the true effect sizes are obtained from a universe of distribution of true effects with a mean and variance and thus the weights are given by:

$w_{i}^{*}$= $\frac{1}{s_{i}^{2}+\tau^{2}}$

where $\tau^{2}$ is measured as between study heterogeneity and the pooled-effect size is given by:

$$\hat{\theta}=\frac{\sum_{i=1}^{I} \hat{\theta}_{i}w_{i}^{*}}{\sum_{i=1}^{I} w_{i}^{*}}$$

The value of $\tau^{2}$ is estimated using DerSimonian-Laird estimator (21).

# References

1. Briones Carvajal JR, Naimi MF, Zhang L, Emmenegger U. Real-world comparison of abiraterone (A) versus enzalutamide (E) for first-line therapy of metastatic castration-resistant prostate cancer (mCRPC). J Clin Oncol (2021) 39(6_suppl):133.

2. López-Campos F, Lorente D, Llacer Perez C, Ramirez-Backhaus M, Peleteiro P, Gomez-Iturriaga A, et al. Evaluation of PSA progression after initiation of enzalutamide or abiraterone: Real-world data on metastatic castration-resistant prostate cancer (mCRPC). J Clin Oncol (2021) 39(15_suppl):5024.

3. Soleimani M, Zou K, Sunderland K, Struss W, Eigl BJ, Nappi L, et al. Effectiveness of first-line abiraterone versus enzalutamide among patients≥ 80 years of age with metastatic castration-resistant prostate cancer: A retrospective propensity score–weighted comparative cohort study. Eur J Cancer (2021) 152:215–22.

4. Tagawa ST, Ramaswamy K, Huang A, Mardekian J, Schultz NM, Wang L, et al. Survival outcomes in patients with chemotherapy-naive metastatic castration-resistant prostate cancer treated with enzalutamide or abiraterone acetate. Prostate Cancer Prostatic Dis (2021) 24(4):1032–40.

5. George DJ, Tagawa ST, Lechpammer S, Russell D, Hong A, Mardekian J, et al. Overall survival (OS) in men with chemotherapy-naïve metastatic castration-resistant prostate cancer (mCRPC) receiving bicalutamide (BIC) followed by enzalutamide (ENZA) or abiraterone (ABI). J Clin Oncol (2020) 38(6_suppl):40.

6. Ramaswamy K, Lechpammer S, Mardekian J, Schultz NM, Huang A, Wang L, et al. PD15-11 Survival rates and economic outcomes in chemotherapy-naïve metastatic castrate-resistant prostate cancer patients treated with abiraterone acetate or enzalutamide. J Urol (2019) 201(Suppl 4):e242–3.

7. Scailteux L-M, Campillo-Gimenez B, Kerbrat S, Despas F, Mathieu R, Vincendeau S, et al. Overall survival among chemotherapy-naive patients with castration-resistant prostate cancer under abiraterone versus enzalutamide: A direct comparison based on a 2014–2018 French population study (the SPEAR cohort). Am J Epidemiol (2021) 190(3):413–22.

8. Chowdhury S, Bjartell A, Lumen N, Maroto P, Paiss T, Gomez-Veiga F, et al. Real-world outcomes in first-line treatment of metastatic castration-resistant prostate cancer: The Prostate Cancer Registry. Target Oncol (2020) 15(3):301–15.

9. Cesca MG, Silveira MT, Pandolfi NC, Oliveira TB, Rinck JA, Da Costa AAB, et al. Comparison of enzalutamide versus abiraterone in castration-resistant prostate cancer before docetaxel: Results of a propensity score-matched analysis. J Clin Oncol (2019) 37(15_suppl):e16540.

10. Komura K, Fujiwara Y, Uchimoto T, Saito K, Tanda N, Matsunaga T, et al. Comparison of radiographic progression-free survival and PSA response on sequential treatment using abiraterone and enzalutamide for newly diagnosed castration-resistant prostate cancer: A propensity score matched analysis from multicenter cohort. J Clin Med (2019) 8(8):1251.

11. Schoen MW, Carson KR, Eisen SA, Bennett CL, Luo S, Reimers MA, et al. Survival of veterans treated with enzalutamide and abiraterone for metastatic castrate resistant prostate cancer based on comorbid diseases. Prostate Cancer Prostatic Dis (2023) 26(4):743–50.

12. Chen HK, Su PJ, Wang YL, Chang KC, Su YL, Chang PH, et al. Long‐term use and risk of major adverse cardiac events: Comparing enzalutamide and abiraterone in chemotherapy‐naïve patients with metastatic castration‐resistant prostate cancer. Int J Cancer (2023) 152(6):1191–201.

13. An H, Schoen MW, Rider J, Schorer AE, Ken J, Chen L, et al. Comparative effectiveness of abiraterone and enzalutamide in the first-line treatment of metastatic castration-resistant prostate cancer (mCRPC): A retrospective cohort study in a large database of deeply curated EHR real-world data (RWD) from community oncology practices in the US. J Clin Oncol (2023) 41(6_suppl):175.

14. Li J-R, Wang S-S, Chen C-S, Yang C-K, Lu K, Cheng C-L, et al. Efficacy of Novel Hormone Agents in the Treatment of Metastatic Castration-resistant Prostate Cancer: A Real-world Retrospective Study. Anticancer Res (2022) 42(10):4857–66.

15. Alkan A, Güç ZG, Gürbüz M, Özgün G, Değirmencioğlu S, Dogan M, et al. Enzalutamide versus Abiraterone Acetate as first-line treatment of castration resistant metastatic prostate cancer in geriatric (≥75) patients. J Mens Health (2021) 17(4):1–7.

16. Marar M, Long Q, Mamtani R, Narayan V, Vapiwala N, Parikh RB. Outcomes among African American and non-Hispanic white men with metastatic castration-resistant prostate cancer with first-line abiraterone. JAMA Netw Open (2022) 5(1):e2142093.

17. Uchimoto T, Komura K, Fukuokaya W, Kimura T, Takahashi K, Nishimura K, et al. Early prostate-specific antigen (PSA) change at four weeks of the first-line treatment using abiraterone and enzalutamide could predict early/primary resistance in metastatic castration-resistant prostate cancer. Cancers (Basel) (2021) 13(3):526.

18. Baillie K, Mueller T, Pan J, Laskey J, Bennie M, Crearie C, et al. Use of record linkage to evaluate treatment outcomes and trial eligibility in a real‐world metastatic prostate cancer population in Scotland. Pharmacoepidemiol Drug Saf (2020) 29(6):653–63.

19. Oruç Z, Kaplan MA, Karaağaç M, Özyurt N, Tatlı AM, Kaya AO, et al. Efficacy and tolerability of current treatments for hormone-refractory prostate cancer patients with visceral metastases. Future Oncol (2021) 17(13):1611–24.

20. Mantel N, Haenszel W. Statistical aspects of the analysis of data from retrospective studies of disease. J Natl Cancer Inst (1959) 22(4):719–48.

21. DerSimonian R, Laird N. Meta-analysis in clinical trials. Control Clin Trials (1986) 7(3):177–88.
